# Perceived Community Support, Users’ Interactions, and Value Co-Creation in Online Health Community: The Moderating Effect of Social Exclusion

**DOI:** 10.3390/ijerph17010204

**Published:** 2019-12-27

**Authors:** Wenlong Liu, Xiucheng Fan, Rongrong Ji, Yi Jiang

**Affiliations:** 1 College of Economics and Management, Nanjing University of Aeronautics and Astronautics, Nanjing 211106, China; jirongrong94@163.com (R.J.); m18015957645@163.com (Y.J.); 2 School of Management, Fudan University, Shanghai 200433, China; xcfan@fudan.edu.cn

**Keywords:** online health community, perceived community support, users’ interaction, co-created value, continuous participation intention, social exclusion

## Abstract

Online health communities (OHCs) face the same problem as other social media platforms in terms of decreasing activity and user attrition. Drawing upon organizational support theory, this study explores how perceived community support affects user interactions and value co-creation which in turn influence their continuous participation. OHCs act as both health knowledge-sharing platforms and important social media for patients, and thus, interpersonal interactions in OHCs are categorized into health-related and general topic interactions. Considering the identity of patients, this study also examines the moderating effect of user-perceived social exclusion on the relationship between community support and user interaction. A total of 292 valid samples from a diabetic patient community in China were used to examine the proposed hypotheses through structural equation modeling. The results show that: (1) Community support has a positive effect on health topic and general topic interactions; (2) both types of interactions have significant positive effects on users’ perceived functional and social values, while general topic interaction is also related positively to users’ perceived affective value; (3) perceived functional value can result directly in continuous participation, while perceived social value contributes indirectly to continuous participation intention through perceived affective value; and (4) users perceived higher social exclusion are more influenced by community support to participate in health topic interactions than those who perceived lower social exclusion, while no significant difference in general topic interactions between two groups. The results of this study can provide implications for both researchers and practitioners.

## 1. Introduction

In recent years, online health communities (OHCs) have become a useful channel for people to seek and share health-related information. According to the report by the Pew Research Center, 59% of American adults have experience in searching for online health information and 35% of have searched for medical solutions through the Internet for themselves or people close to them who might have a disease [1]. The boom of OHCs worldwide is being accelerated by advancements in Internet technology, leading to its evolution into various forms. The most representative forms are patient-to-doctor communities and patient-to-patient communities of special interest. The patient-to-doctor communities serve as a bridge between patients and doctors online and provide platforms for doctors to deliver information and consultations to patients [2]. The patient-to-patient communities connect patients with the same diseases and enable them to share information on their condition, treatments as well as offer advice and emotional support to others in the community [3]. In patient-to-doctor communities, managerial issues are concentrated generally on achieving mutual benefits to promote the development of OHCs, such as alleviating information asymmetry between doctors and patients, improving incentive mechanisms to ensure doctors and patients’ continuous participation, optimizing service delivery process or adjusting prices of premium doctor-patient interactions [4]. By contrast, in patient-to-patient communities, which have no inequality in personas (like doctors and patients in patient-doctor OHCs) and financial exchange among users, members’ active participation is acknowledged as essential for the sustainability of the communities [5]. Patient-to-patient OHCs, as a patient communication platform, face the same problems as other social media networks where the turnover and migration happen [6]. These problems drive many researchers and practitioners of OHCs to attempt to factors that affect users’ continuous participation in patient-to-patient OHCs.

Previous studies have explored users’ intentions in participating in OHC interactions from both intrinsic and extrinsic motivations. Knowledge self-efficacy, altruism, empathy, sense of self-worth, face concern (an important concept in Chinese culture; representing the need for esteem via knowledge sharing in the context of online communities), perceived vulnerability, and health severity are acknowledged as intrinsic factors that affect users’ information sharing behaviors [7,8,9]. As extrinsic factors, reciprocity, reputation, perceived informational, social, emotional support from other members are determined to have important influence on users’ information sharing intention [7,8]. Meanwhile, human-computer interaction-related factors have been addressed in other online communities (e.g., brand community) to study user participation behavior [10]; however, these studies have merely emphasized the aspect of information technology and system design. Recently, researchers have introduced organizational support theory into the online community and verified that user-perceived community support can lead to high intentions of knowledge contribution and commitment [11,12]. Organizational support refers to organization members’ general beliefs regarding the extent to which the organization values their contribution and cares about their well-being [13]. Individuals’ perceived organizational support will motivate them to reciprocate the organization with affective commitment and organizational spontaneity [14]. In the context of online communities, perceived community support reflects users’ views on the extent to which the online community facilitates their communication and recognizes and rewards their contribution, which will in turn influence users’ continuous participation [11].

According to the organizational support theory, members tend to exhibit organizational citizenship behaviors (OCB) if they perceive support from the organization [12,15]. In an online community, such behaviors are called online community citizenship behavior (OCCB) and benefit the community as a whole [16]. OCCBs involve different types of community participation behaviors, such as information sharing, knowledge contribution, topic discussion, community interaction, among others [7,8,10,12,17,18,19,20,21,22]. Whether sharing, discussing, integrating, commenting, or reposting, users generally engage in social or personal interactions [12]. These interactions are the key factors that allow communities to survive and thrive [23]. OHCs, as a specific type of online community, also face the challenge of user turnover [6]. OHCs should provide convenience to support individuals to interact with experientially similar others in regard to both health issues and social support [4]. Though perceived community support is related positively to users’ knowledge contribution and their commitment to the online community [11,12], the influencing mechanism has not yet been discussed adequately, especially in patient-to-patient OHCs. 

Previous studies have revealed that user interactions can create benefits or values to them, which then result in commitment and loyalty to the online community [20,24]. On this basis, this study proposes a framework to examine the relationships among community support, user interactions, perceived co-created value, and continuous participation intention in OHCs. As health knowledge-sharing platforms and important social media for patients, topics discussed in OHCs can be categorized as either general (public) or specific (private) [7], also referred to as on-topic and off-topic discussion [17]. Both types of interpersonal interactions are believed to contribute to improving users’ relationships and their attachment to the community [10,17]. Thus, in this study, interpersonal interactions are also sorted into two categories, namely, health-related topic interactions and general topic interactions. Personality traits and identity of patients may result in social interaction anxiety in the real world, which in turn makes individuals perceive higher social exclusion [19,25]. Community support may exert a more significant effect on encouraging this group to interact actively with others in OHCs. Thus, the moderating role of perceived social exclusion between community support and user interactions is explored in this study. Several theoretical and practical implications are discussed based on the results of the empirical analysis. 

## 2. Literature Review

### 2.1. User Participation in Online Communities

User participation in online communities has attracted wide attention from practitioners and researchers alike because active participation is considered essential for the sustainability of a community [5]. Community participation behavior has been investigated mainly from two perspectives: knowledge sharing and user interaction [4,7,8,10,18,20,21,26]. Knowledge sharing as explored in previous studies focused primarily on users’ motivation for contributing to online communities [19]. Personal factors (e.g., empathy, enjoy helping, self-efficacy, prosocial value orientation, etc.), technological factors (e.g., community artifacts, perceived usability, etc.), and contextual factors (e.g., trust, community interest, identification, normative influence, norm of reciprocity, shared vision, and outcome expectations) can be concluded as influencing factors that motivate users to contribute their special expertise or share useful information to help others solve problems [19,27,28,29,30,31]. Knowledge sharing can be considered a behavioral consequence stimulated by internal and external factors. Though some user interaction-related studies have emphasized users’ intrinsic and extrinsic motivation to participate in the community interaction, more studies have concentrated on the types of interaction that can be summarized as product-information interaction, human-computer interaction, and person-to-person interaction [10,32]. Product-information interaction refers to community users exchanging information and personal experiences of a product or discussing specific issues, such as how to solve a technical problem in product or service use [20,33]. Human-computer interaction underlines community interactivity, which is linked closely to information technology [10]. Community interactivity generally refers to the degree to which community users can interact with others or the platform [20]. The higher the interactivity, the more effective the communication [20,33]. Person-to-person interaction is also called interpersonal or social interaction [10,34,35]. In an online community, a high level of interpersonal interaction creates a harmonious atmosphere of active communication and mutual aid [10,36]. Individuals not only interact with others to exchange opinions or information on specific issues but also to share values and establish relationships with other members of the community [4,34,35]. Effective interactions can lead to community commitment, which in turn, affects users’ behavioral intention and loyalty [11,20]. Table 1 summarizes some of the studies that focused on user participation in various online communities. 

According to Table 1, when discussing user participation in an online community, most of the studies emphasize users’ knowledge sharing behavior and only a few focusing on other perspectives such as community interactions. Researchers have also focused on investigating users’ intrinsic and extrinsic motivations to contribute their knowledge. In other words, knowledge sharing behavior is generally considered a consequence evoked by both individual and environmental factors. On the contrary, the user’s interaction in a community is believed to be fundamental in building social cohesiveness, shared value, and expectations of collaborative interpersonal relationships, which in turn increase users’ attachment to the community [17]. Compared to simple knowledge sharing, interpersonal interactions reflect not only the initiative and enthusiasm of the users for seeking information but also the two-way communication among the community users, which is more effective in the construction of harmonious community relations and in the strengthening of users’ community identification and loyalty [10,35]. Though most community interaction studies have focused on its considerable role in shaping users’ long-term participation behavior, some researchers have also shed light on the determinant of user interactions. Community environment and user-perceived community support, namely, support for member communication, recognition for contribution, and freedom of expression, will affect user participation and contribution intention [11,12,39,40]. Studies also show that individual characteristics and intrinsic motivations, such as neuroticism, extraversion, narcissism, and self-disclosure, among others, influence an individual’s organization citizenship behavior, such as knowledge contribution in the online community [11,19,38,39,41]. 

### 2.2. Interpersonal Interactions in Online Health Communities

Online communities are regarded as both communication platforms and social networks where people sharing the same interest can contribute their knowledge and participate in interactive activities with one another [11,42]. An OHC is a specific type of online community through which the health-related topics are discussed [5]. Users in OHCs not only share specific information, such as medical treatments, personal health conditions, and medical experiences, but also share some general information, such as hospital or doctor information [7]. In addition to these kinds of on-topic interactions (health-related discussions), users also initiate off-topic interactions (other discussions, such as private information, everyday life, or hot social topics) which are regarded as a double-edged sword to the community. Off-topic discussions can be distracting and may be off-putting to new users whose initial expectations are likely to be disappointed; however, it can also provide more opportunities for users’ self-disclosure and friendship [17]. If designers discourage off-topic discussions, they might lose people who like to talk with others like themselves. The discouraging off-topic discussion also may annoy old-timers, who have gotten to know each other. Thus, the choice that community designers make on whether to allow off-topic discussion can influence who joins the community and who stays [43]. For people with a high level of social isolation, online communities provide them a channel for anonymous communication, which facilitates self-disclosure and self-presentation [25]. In a health-related context, patient’s relationships with others in the real world can become strained [44]. In this regard, off-topic interactions among users can attract patients who have an unsatisfied social need or are suffering from social isolation. Considering patients’ particular need to obtain social support and reconnect with others, most OHCs do not discourage off-topic discussions and even set up separate modules (e.g., Mood Inn and Partying & Friending in bbs.tnbz.com) for users to initiate and participate in activities or discussions other than health topics. In this study, both health-related and other topical discussions are considered as indispensable components of the interpersonal interactions among OHC users and are named respectively as specific (health-related) topic interaction and general topic interaction. 

## 3. Hypotheses Development

### 3.1. Perceived Community Support and User Interactions

Online communities have rapidly evolved into an important form of human organization [45]. However, despite the rapid growth of the online community, previous studies have shown that users in an online community have a higher rate of turnover [11,45]. OHCs face the same problems as other online communities where user loss happens unless tailored content and appropriate socialization are supported [6]. Community operators exert considerable effort to retain their users by motivating participation, including constantly optimizing the community structure and features as well as organizational policies [43]. Researchers advocate a supportive organizational climate, which refers to openness, honest communication, participativeness, trust, mutual help, and cooperation, have notable effects on organizational citizenship behavior and the effectiveness of communication and cooperation [15,46]. Online communities are social entities composed of people and their relationships, and their success depends on users’ OCCB that benefit the community as a whole [16]. Thus, structures and policies of online community cannot be designed without a thorough understanding of users’ citizenship behaviors and the determinant of those behaviors. Community users’ contribution can be viewed as a critical form of OCB and is related positively to their perceived community support [12]. User-perceived community support, including perceived support for member communication, perceived recognition for contribution, and perceived freedom of expression, has been determined to have considerable effects on user’s commitment [11]. Social interactivity, which is regarded as a dimension of community environment, plays a very important role in motivating users’ participation behavior [40]. Community norm, as a behavioral regularity that refers to a rule for what should be done, accepted, and internalized by the community members, has a significant effect on a user’s intention to reciprocate [47]. OHC studies also found that feedback, rewards, and reputation systems of the community can encourage user participation and contribution [7]. Providing technical support for effectively storing, organizing, and modifying knowledge can lead to higher perceived usefulness and positive attitude to participate [48]. Successful community system design and policies guide users’ interactions and facilitate the sense of togetherness [5]. Hence, the following hypothesis is proposed:

**Hypothesis** **1.** *Perceived community support positively affects users’ interactions, including (a) health-related topic interactions and (b) general topic interactions*.

### 3.2. Users’ Interaction and Value Co-Creation

Customer-to-customer interaction (CCI) has been noted in the service context decades ago and is considered an important aspect of the service experience as well as a driver of customer satisfaction and loyalty [49]. CCI is believed affect key concepts of service marketing, such as the service profit chain and service productivity because it can add value for customers [50]. Such interactions occur offline and online and create opportunities for increasing participatory engagement [51]. In the Web 2.0 context, interactions between users are the core of social media or platforms and are regarded the key part of value creation [50]. Spending time with peers and significant others, interacting and connecting with strangers, or simply being co-present as part of a large collective may not link directly to immediate benefits, but the value is formed in the customer-to-customer co-creation [52]. Customers’ voluntary participation is a sign of value co-creation. 

Researchers have been exploring the notion of ‘perceived value’ or ‘consumer value’ for decades. Previous studies undertaken within the areas of tourism, events, and hospitality services have unveiled the positive effect of customer-to-customer co-creation on individual value outcomes, such as well-being and relationships [53,54]. However, because perceived value is highly subjective to each individual, researchers and practitioners have sought to identify specific types of value to determine the customers’ needs and how these needs can be fulfilled [55]. Sheth et al.’s (1991) multi-dimensional value model (social, emotional, functional, epistemic and conditional) has been adopted in many studies that explore how each type of value influences consumers’ decision-making in different situations [56]. In Customer-Dominant logic, customers co-create values (affective, functional, social, and network value) by interacting with each other and integrating their resources [52]. A study of customers’ value co-creation crossing nine online brand communities uncovered social networking and community engagement as two important categories of customer-to-customer value [57]. The study pointed out that social networking practices, such as ‘welcoming’, ‘empathizing’, and ‘governing’ help to create, enhance, and sustain social and moral bonds among community members, which often extend outside the community itself. Through practices, such as ‘staking’, ’milestoning’, ‘badging’, and ‘documenting’, community members reinforce their engagement with and increase their social capital within the community.

Drawing on the above literature, functional, social, and affective values can be generated as the three primary and inclusive categories of user-to-user co-created values in online communities. Functional value can be understood as the cognitive value related to efficiency or the excellence value gained from advice or support offered by other users [58]. Social value refers mainly to group membership and social identity. Affective value refers to users’ hedonic or emotional perceptions, such as enjoyment, belongingness, or happiness [56,59]. User’s community engagement, such as information sharing and user-to-user interaction, influences their perceptions of benefits including learning, social and hedonic benefits [20]. Community members discuss specific topics to increase their understanding or knowledge of the issues that interest them [32]. Such in-depth interactions can, in turn, increase the likelihood of solving related problems. Connections among users based on the interactions on their shared interest can make their social relationship more meaningful and make them feel good about themselves [60,61]. A wide range of topics can be discussed in online community, thereby enabling members to expand their social network through interactions [32]. In OHCs, both specific and general knowledge are generated because OHCs are a channel for patients to discuss health and other social or emotional topics, especially for patients who perceive high levels of social isolation [7,25]. In other words, the OHC also serves as social media network for patients to exchange various knowledge and social support. All types of social interactions occurring in the online community can influence user-perceived learning benefits, group identification, relationships with others, wellbeing and community attachment [10,17,20,35]. Thus, the following hypotheses are proposed:

**Hypothesis** **2.** *Health-related topic interactions positively influence a user’s perceived (a) functional value, (b) social value, and (c) affective value of the OHC*.

**Hypothesis** **3.** *General topic interactions positively influence a user’s perceived (a) functional value, (b) social value, and (c) affective value of the OHC*.

Affective value relates to personal satisfaction, personal growth, and a sense of joy and pleasure [52]. Users in the online community achieve knowledge gathering and learning according to others’ advice, information, and expertise. Meanwhile, online communities serve as a means for fulfilling personal as well as social needs. Once the users are satisfied with these functional and social benefits, it can result in a sense of belonging, happiness, enjoyment as well as attachment and commitment to the community [17,20,24,52]. Users’ perceived social support through interpersonal interactions also can promote their subjective well-being [16]. Particularly in OHCs, patients seeking and offering informational and social support to each other can satisfy their need for love, belonging, esteem, and self-actualization [7]. Thus, in this study, we propose the following hypothesis:

**Hypothesis** **4.** *Users’ perceived (a) functional value and (b) social value through interactions in an online health community can contribute to their perceived affective value*.

### 3.3. Perceived Co-Created Value and Continuous Participation Intention

Co-created values are the value construct appraised by individuals with regard to the meaningfulness of a target (product or service, further referred to as service) based on what is contributed and what is realized through the process of co-creation [24]. Users’ co-created values in an online community can be functional, social or/and affective benefits as discussed above. From the perspective of social exchange, perceived benefits are crucial factors that drive continuous supportive exchange because of individuals who perceive a moral obligation to pay back the benefits to the other party [62]. Within this logic, individuals who perceived community support experience a sense of indebtedness and thus, are propelled to reciprocate by making contributions to the organization [12]. People who share online believe in reciprocity and expect that interactions will be available in the future, specifically interactions related to knowledge provision and reception [21]. Generally, reciprocity is perceived as a norm in the community, and individuals consciously or unconsciously feel that reciprocal contributions are expected by others in the community, and thus would intend to engage in reciprocal interactions in the community [47,63].

From the perspective of organization related theory, individuals who perceive benefits from the organization intend to engage in organizational citizenship behaviors [12]. Community members who obtained informational and social support from the community are found to be more likely to maintain a long-term relationship with the community [64]. These kinds of support can lead to higher community identification and subjective well-being, which in turn contribute to users’ OCCB [16], such as offering support to other members [39]. As members in the online community generally share the same interest or vision, their perceived similarity and identity lead to specific bonds that will result in the attachment to the community and make them participate more actively in on-topic and off-topic discussions [17]. Particularly in the online health community, users’ altruism and empathy to others will have a positive influence on the others’ intention to contribute [8]. Moreover, continuous participation also can fulfill the users’ social and emotional needs [38]. In conclusion, user-perceived values in the community interaction, including functional, social and affective values, make them loyal to the community and propel to initiate or participate more actively in the community interaction. Thus, this study set up the following hypotheses:

**Hypothesis** **5.** *Users’ perceived (a) functional value, (b) social value, and (c) affective value through interactions in the OHC will lead to continuous participation intention*.

### 3.4. Role of User-Perceived Social Exclusion

Social exclusion is the general perception of an individual who feels isolated, excluded, rejected, or ignored by others [65]. As a perceived deficit in belongingness, social exclusion causes strong negative emotions, such as loneliness, jealousy, depression, and anxiety, which threatens an individual’s fundamental psychological needs, such as the need for belonging, self-esteem, control, and a sense of meaningful existence [66,67]. Social exclusion can limit an individual’s interactions with others, leading to the perceived inability of establishing or maintaining positive interpersonal relationships and resulting in negative emotions, such as anxiety, which refers to excessive fear of potentially embarrassing or humiliating situations [65,68]. Individuals who perceive social exclusion may pay more intention to potential sources of social connection [69]. Online social media networks, which are effective tools to acquire social connection, have been found to be associated closely with responses to social exclusion. Individuals feel less socially anxious when socializing online than when communicating face-to-face, and their suffering from exclusion can be relieved [66]. 

In the health-related setting, interactions and relationships with family and friends can become strained and appropriate support can be withdrawn because of extensive illness-related concerns among caregivers and supporters [70]. People who lack positive relationships often experience loneliness, guilt, depression, and anxiety, and feel the restriction of access to resource structures necessary for social belonging [44]. Given that social platforms can serve as a convenient platform for acquiring social connection, it is reasonable to suppose that when perceiving social exclusion, individuals may be primed to think about online communities as a means to meet fundament human needs [69]. In online community, both community factors (environmental factors) and individual factors (personal characteristics) have been shown to influence user participation behaviors [38]. Drawing upon the literature, this study suggests that the supportive community climate will positively affect users’ interactions. Patients with high levels of social exclusions seek a variety of online social support than those patients with lower levels of social exclusion [44]. Moreover, online knowledge community-related studies have shown that intrinsic motivations would moderate user’s knowledge sharing behavior [19]. Thus, this study proposes the following hypotheses:

**Hypothesis** **6.** *Users’ perceived social exclusion will moderate the relationships between community support and user interactions, including (a) health-related topic interactions and (b) general topic interactions*.

## 4. Methods

### 4.1. Research Model and Measurement 

**Figure 1:**
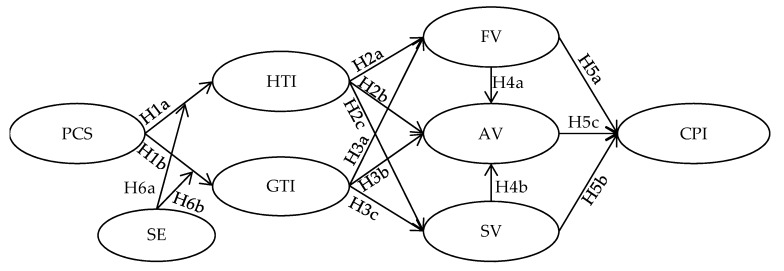
Research model.

This study seeks to investigate the effects of user-perceived community support on their continuous participation in OHCs by integrating user-to-user interaction and value co-creation and considering the user’s social exclusion. The research model is presented in Figure 1. This study proposes several hypotheses that are arranged in four categories: (1) perceived community support to user interactions; (2) user interactions to value co-creation; (3) co-created value for continuous participation; and (4) the moderation of users’ perceived social exclusion. In Figure 1, PCS indicates perceived community support; HTI indicates health topic interaction; GTI indicates general topic interaction; FV indicates functional value; SV indicates social value; AV indicates affective value; CPI indicates continuous participation intention; SE indicates social exclusion.

A survey questionnaire is developed based on the model. To measure each construct, questions were adapted from validated instruments used in the previous studies and modified to fit our research context. We adapted items for perceived community support from Yan et al. [7] and Yang et al. [11], items for health-related topic interactions and general topic interactions from Yan et al. [7] and Fiedler and Sarstedt [17], items for user-perceived co-created value, namely functional value, social value, and affective value from Yan et al. [7], Kuo and Feng [20], and Lin et al. [39], items for continuous participation intention from Ye et al. [12] and Fang and Zhang [71], and items for social exclusion from Zhang et al. [40] and Yao et al. [44]. Table 2 shows a list of the items for all constructs. All items are measured with a five-point Likert-type scale (1 = strongly disagree, 5 = strongly agree). 

### 4.2. Samples

To test our hypotheses, we collected data from the Sweet Home (http: bbs.tnbz.com). Sweet Home, which has more than 150,000 members, focuses on diabetes and provides a communication platform for diabetic patients to discuss both diabetes treatment and other health or non-health related topics. We posted a survey invitation and questionnaire link in Sweet Home. To encourage community users to participate in our survey, 6 RMB was promised to be remitted into each respondent’s Alipay account according to the phone number provided in the survey questionnaire. The data collection procedure took place from 15 April 2019 to 15 May 2019. A total of 319 responses were received. After discarding 27 incomplete questionnaires, 292 samples were used for the final analysis. 

Table 3 shows participant demographics. A total of 46.2% of the participants were males and 53.8% were female. Most of the respondents had ages ranging from 26–45 years old (76.4%) and have university or higher educational background (66.1%). In terms of monthly income, 66.4% earn more than 5000 RMB per month. Most of the participants have been using this OHC for more than three months, specifically, 47.3% for three months—one year and 43.8% for more than one year. In summary, the samples are approximately in a normal distribution.

### 4.3. Test of Reliability and Validity

The reliability, convergent validity, and discriminant validity of the scales were examined by using confirmatory factor analysis (CFA) based on AMOS 21.0. Table 4 shows that the measurement model demonstrated a good model fit (χ^2^/df = 2.263, GFI = 0.905, RFI = 0.919, TLI = 0.953, CFI = 0.967, RMR = 0.036, RMSEA = 0.066). The factor loadings in the measurement model all exceeded 0.7 except for GTI2 (factor loading is 0.697) which is also very close to 0.7. Moreover, all the values of reliability (Cronbach’s α) and composite reliabilities are greater than 0.7, thereby demonstrating the convergent validity of our measures [72,73].

The square root of all average variance extracted (AVEs) is higher than the correlations between the target variable and any of the other ones (see Table 5), which demonstrates the discriminant validity of the measures. 

Potential biases resulting from common-method variance (CMV) were checked because self-reported data were used in this study. First, Harman’s one-factor analysis as a statistical remedy is conducted. The CFA results showed that the one-factor model (χ^2^/df = 12.216, GFI = 0.490, RFI = 0.560, TLI = 0.581, CFI = 0.621, RMR = 0.095, RMSEA = 0.196) exhibited a worse fit than our measurement model. In addition, all items are entered into an unrotated exploratory factor analysis to determine whether a single factor emerges or a single factor accounts for the majority of the variance. The results show that the first component with the largest eigenvalue explains 25.12% of the variance, which does not exceed 50% of all variances [74]. The above results indicate that the common-method bias (CMB) is not an issue in this study. 

## 5. Hypothesis Test

### 5.1. Summary of Path Analysis Results

**Figure 2:**
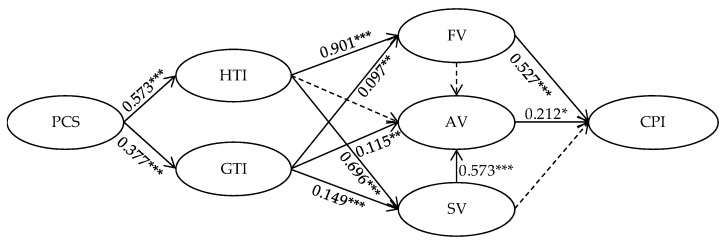
Path coefficient of the hypothesis model. * *p* < 0.05; ** *p* < 0.01; *** *p* < 0.001.

To test the hypotheses proposed in this study, structural equation modeling analysis is conducted on AMOS 21.0. The result presents a good model fit (2/df = 1.984, GFI = 0.925, RFI = 0.937, TLI = 0.968, CFI = 0.977, RMR = 0.030, RMSEA = 0.058), and path coefficients between each pair of variables in the structure model are shown by Figure 2.

Hypotheses 1a,b propose that a supportive community climate positively affects both health topic interaction and general topic interaction between community users. As shown in Figure 2, the path coefficients of supportive community climate on health topic interaction (β = 0.573, *p* < 0.001) and general topic interaction (β = 0.377, *p* < 0.001) are both positively significant, which supported hypothesis 1a,b. Hypotheses 2a–c propose the positive relationships between health topic interaction and users’ co-created values, including functional, social, and affective value. The path coefficients show that users’ health-related topic interactions are positively related to their perceived functional value (β = 0.901, *p* < 0.001) and social value (β = 0.696, *p* < 0.001), but not significantly related to affective value (β = 0.275, *p* > 0.05). Thus, Hypotheses 2a,c are supported, while Hypothesis 2b is not supported. Meanwhile, Hypotheses 3a–c propose the positive relationships between general topic interaction and users’ co-created values. According to the path coefficients, the general topic interactions are positively related to users’ perceived functional value (β = 0.097, *p* < 0.01), social value (β = 0.149, *p* < 0.001), and affective value (β = 0.115, *p* < 0.01), which supported the Hypothesis 3a,b, and 3c. Hypotheses 4a,b propose that users’ perceived functional value and social value through interactions in an online health community can contribute to their perceived affective value. The result shows that perceived social value positively influence users’ affective value, which supported hypothesis 4b (β = 0.573, *p* < 0.001). However, the path coefficient between perceived functional value and affective value is not significant (β = 0.098, *p* > 0.05), which indicates hypothesis 4a is not supported. Hypotheses 5a–c propose the positive relationships between users’ co-created value and their continuous participation intention. The path coefficients show that both functional value (β = 0.527, *p* < 0.001) and affective value (β = 0.212, *p* < 0.05) are positively related to users’ continuous participation intention, but the relationship between social value and continuous participation intention is not significant (β = 0.105, *p* > 0.05). Thus, Hypotheses 5a,c are supported, while 5b is not.

As previous studies suggest that users’ perceived social value via participating in community interactions can make them loyal to the community, this study believes there should be some relationship between users’ perceived social value and their continuous participation intention. Since path analysis result does not show a direct effect between these two variables, an indirect effect analysis is conducted based on the Bootstrap Method (2000 bootstrap samples, 95%PC). As shown in Table 6, although users’ perceived social value does not have a significant direct effect on continuous participation intention (β = 0.105, *p* > 0.05), it exerts an indirect effect (β = 0.121, *p* < 0.05) through affective value, and a significant total effect (β = 0.226, *p* < 0.01), which, to some extent, proved the contribution of perceived social value on user’s continuous participation intention.

### 5.2. Moderating Effect of Social Exclusion

**Figure 3:**
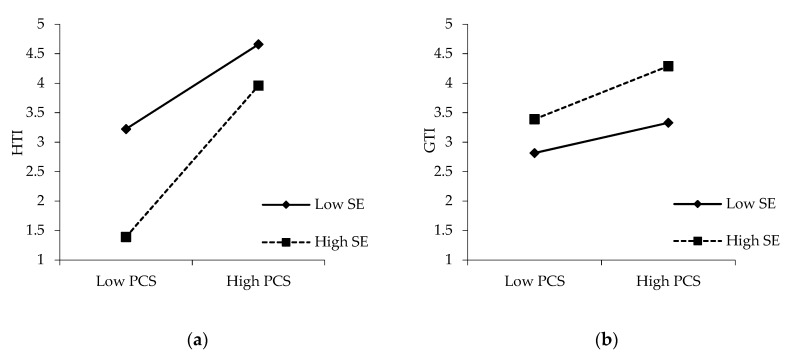
Moderating effects of perceived social exclusion between (**a**) community support and health topic interaction; (**b**) community support and general topic interaction.

Hypotheses 6a and 6b propose that user-perceived social exclusion will moderate the relationships between supportive community climate and user participation in both health-related and general topic interactions. To test these hypotheses, this study conducted multi-group path analyses after dividing the samples into two groups based on the mean of the perceived social exclusion. According to the result, the change of χ^2^ value is statistically significant (∆χ^2^ = 37.952, *p* < 0.001) when constraining the path between supportive community climate and health topic interactions. The slopes in Figure 3a indicate that users who perceive a higher level of social exclusion are more easily encouraged by the community’s supportive climate to participate in health-related topic interactions (β = 0.789, *p* < 0.001, *N* = 173) than those who perceive a lower level of social exclusion (β = 0.736, *p* < 0.001, *N* = 119). Thus, hypothesis 6a is supported. However, when constraining the path between supportive community climate and general topic interactions, the change of χ^2^ value is not significant (∆χ^2^ = 3.271, *p* > 0.05). Figure 3b shows that the difference between the two slopes is unremarkable. Therefore, hypothesis 6b is not supported. 

## 6. Discussion

By adopting the organizational support theory and the concept of organizational citizenship behavior into the online health community, a virtual organization context, this study proposed a framework to understand OHC users’ continuous participation intention from the perspective of the community’s support, user interactions, and value co-creation. This framework considers organizational support as one of the extrinsic motivations of user participation. However, this framework does not ignore the role of the user’s intrinsic motivation, namely perceived social exclusion, which is a representative trait of patients who are generally the users of OHCs. Based on the results of the empirical analysis, this study generates several theoretical and practical contributions.

### 6.1. Theoretical Implications

First, positive relationships can be observed between the OHCS’ supportive climate and users’ interactional behaviors. This result echoes that of previous studies on users’ online participation behavior, which suggests that environmental factors influence a user’s intention to engage in the community [12,38]. Specifically, perceived community support is found to affect strongly a user’s knowledge contribution in the online knowledge community [12]. Drawing upon the organizational support theory, individuals who perceive support from an organization tend to exert organizational citizenship behaviors. As a virtual organization, OHCs’ supportive climate can lead to higher interactional behaviors. A previous study revealed that community support, such as recognition, feedback, rewards, etc., is related significantly to community users’ knowledge sharing behavior, including both general and specific knowledge sharing [7]. Similarly, this study found that a supportive community climate contributes to both health and general topic-related interactions. This result indicates that the OHCs are not only a platform for health information sharing but also a medium for patients to discuss other topics in their daily life, which may be limited in the real world because of their health situation. In other words, both health knowledge and social exchanges are expected by users and indeed occur in the OHCs. Moreover, community support can enhance users’ intention to participate in various community interactions. 

Second, according to the results, the positive relationships between user interactions and their perceived values indicate that user-to-user interaction is a value co-creation process. This conclusion was obtained from studies on perceived benefits from community interaction. In the online brand community, product information-related interactions are verified to have a positive influence on perceived learning benefits of brand community members [20]. Correspondingly, this study found that health topic interactions are related positively to users’ perceived functional value of the OHCs. Social value also can be co-created in the health topic interactions among users of online health community. Shared interest and experience sharing behavior enable users’ social relationships to become closer and more meaningful [60]. Users can expand their social networks through interactions [32]. Moreover, an online community is not only a platform for exchanging knowledge and experience but also a space to socialize [20]. General topic interactions, which are also defined as off-topic discussions in some studies, do not focus on certain health or disease issues but rather generate knowledge and information on some other field, such as social issues, daily life, or job skills. This type of interaction is verified to have positive relationships with users’ perceived functional, social, and affective values. If users can interact with others on any idea or questions they posted in the community, they will feel that the community is helpful in increasing their knowledge and solving their problems. This process can also strengthen their connections with other members and make them feel pleased and inspired by the community [20]. In this part, unlike general topic interaction, health topic interaction is not related significantly to affective value directly. General topic interactions are free topic interactions that can not only improve users’ relationships but also appeal to their mood and lead to positive emotions [17]. By contrast, health information exchange is the main objective of individuals who participate in OHCs and a social network is gradually formed through user interactions. Although health topic interaction cannot result directly in affective values, it may enhance users’ bond with the group through the network and their perceived social value. 

Third, this study found that users’ co-created values, namely, functional, social, and affective value, affect their continuous participation behavior directly or indirectly. This result echoes the perspectives of previous studies on customers’ perceived value and continuous intention. Customers’ perceived value is related significantly to their loyalty [24]. In the online community context, users’ perceived value has a positive effect on users’ commitment, contribution intention, and cooperation with others and the community [20,21,35,38]. Users who obtained informational, network, and emotional support can enhance their community identification and willingness to offer support to others in the future [39]. Moreover, in the OHCs, both general and health-related topics can be discussed, making the users more open about their life and health situation that they may feel sensitive and not able to talk about with others in the real world. Once they receive knowledge or help from other users, they will feel indebtedness [47]. According to the equity theory, a desire to reduce the feeling of indebtedness can motivate individuals to reciprocate the efforts of other members in the online health community. Meanwhile, as a group of patients who have the same health problem and strong community identification, they will feel the obligation to maintain the development of the community through continuous participation to create sustainable values for themselves.

This study also found that perceived social exclusion can moderate the effect of supportive community climate on health-related topic interactions but have no moderating effect on general topic interactions. This result, to some extent, consists of studies on social exclusion and online behaviors. A previous study found that intrinsic motivations moderate the effects of knowledge sharing in online communities [19]. The result of this study indicated that no difference exists between higher social exclusion users and lower ones in terms of participating in general topic interactions because these interactions may not be sensitive unlike like health-related topics. However, in terms of health-related topics, users with high-level social exclusion are more encouraged by a community’s support to share their health situation and treatment experience, which they may not be willing to discuss in the real world due to the fear of being isolated or abandoned. The online platform provides users with a convenient channel and anonymous communication, which facilitate self-disclosure and self-presentation [25]. Thus, a supportive community climate has a stronger positive effect on users’ participation in health-related topic interactions than in general topic interactions.

### 6.2. Practical Implications

With the rapid growth of general (multi-disciplinary) and specialized OHCs, operators are driven to improve their user management and motivating strategy to encourage continuous participation, contribution, and commitment among users. The findings of this study suggest that managers of OHCs should recognize the importance of users’ perceived community support in drawing users to participate in community interactions. The OHCs should provide various and convenient means of support for user interactions, such as real-time chatting or discussions, and comments, reposting, and liking the content posted by others, which can not only facilitate user experience communication and health knowledge exchange but also improve the relationships between community users. 

Second, in addition to the platform of health information sharing, OHCs are also a social network for users who have the same identity, namely patients of a particular disease, to share or discuss other issues in their life or the society to meet their social needs. Thus, OHCs should offer various modules to support user interaction on health, emotions, life, hobbies, etc. In doing so, users can perceive the community’s consideration and the freedom of expression that it fosters. Meanwhile, a specified module for health topic interaction can assure users of the quality and efficiency of information acquisition. 

Third, OHCs should recognize the users and offer feedback for their participation, such as provide proper rewards to active users for their efforts, design a reputation system for users’ contributions, provide online or offline health consultations or other events for active users. These efforts will help users perceive recognition, belongingness, and even self-esteem especially for patients who perceive higher social exclusion and are not willing to talk about health problems because they care strongly about others’ attitudes towards them. The community’s support will encourage them to participate in the virtual environment’s health topic interactions and help them reach their informational and social goals. Thus, the online community should provide various types of supports to facilitate user interactions and enhance their perceived value through these interactions. The community’s support and users’ co-created value should be recognized by community operators as critical factors to maintaining users’ continuous participation and the community’s sustainable development.

Last but not the least, OHCs might regularly organize online and offline activities. For example, online prize-winning quiz activities can not only encourage users to learn and share health knowledge but also enhance their engagement with the community. Moreover, the prize itself can motivate users to participate in the community activities. OHCs can also invite health experts to offer free online consultation periodically. This can be regarded as an incentive for current users as well as a means to attract potential users. On the other hand, offline activities can support community members to communicate face to face and thus enhance their relationships between each other. More importantly, offline activities may help individuals who perceive high level of social exclusion to overcome their social anxiety. In conclusion, OHCs should provide various supports, including online and offline, to facilitate both the user-to-user interactions and the user-to-community interactions. We believe that supports and benefits perceived by users will eventually result in affective commitment and continuous participation.

### 6.3. Limitations and Future Research

Despite its theoretical contributions and practical implications, this study still has several limitations. First, in terms of sampling, some difficulties were encountered in delivering the questionnaires and collecting data from the special group of people with health problems. Finally, only 292 valid responses were obtained through online surveys, which may not be sufficient to represent the millions of users of OHCs and may affect the accuracy of the overall judgment on user participation behaviors in the community. In particular, the moderation effect of perceived exclusion on the path between supportive community climate and general topic interactions is not significant. Hence, stating unequivocally that no relationship exists based on the limited samples is difficult. Moreover, the samples were collected from the online community of diabetic patients who are suffering from a chronic disease and some differences may exist between users with different types of diseases, such as chronic diseases or cancers. Thus, future work should collect more samples from different health communities and conduct a comparative study. 

Second, this study considers user perceived social exclusion as a moderating factor and assumes that users who perceive a higher level of social exclusion will be more encouraged by the community’s support. Thus, although the results confirm our hypotheses to some extent, there must be some other factors that moderate the effect of community support on user interactions. For example, the member roles, namely posters and lurkers, are distinguished and found to have a moderating effect on perceived community support and online community users’ commitment [11]. Similarly, perceived health status, poor or good, has been found to moderate the relationship between perceived benefits and personal health information disclosure intention [9]. These findings indicate the possibilities to explore the moderating effects of other factors in future research.

Third, this study examined the relationship between community support and user interactions with focus on the patient-to-patient online health community. Currently, both patient-to-patient and doctor-to-patient online health communities have drawn the attention of researchers. Hence, determining whether community support plays critical roles in both doctor-users’ and patient-users’ continuous participation in the doctor-to-patient community is worth exploring. We believe it will contribute to the development of the online health and medical industries.

Fourth, big data techniques and analyses have been successfully applied to many filed, including healthcare informatics, e-health, and social media users’ behavior [75]. In the future, we will consider the methods and tools of big data to deeply and continuously study the data generated by the users of online health community to understand their participation behavior. 

## 7. Conclusions

As an important social assistance resource for healthcare and medical systems, OHCs are addressing many problems for patients and the entire society. However, the community’s low activity and user turnover rates pose significant problems for OHCs. This study proposed a framework for understanding the importance of supportive community climate in users’ continuous participation based on organizational support and value co-creation theories. Scales were developed based on previous studies and used to measure the constructs in the research model. A total of 292 samples were used to examine the proposed hypotheses. Based on the results, theoretical contributions and practical implications were generated. We believe our study can provide reference for both academic researchers and practitioners in this field. 

## Figures and Tables

**Table 1:** Summary of studies on users’ participation in an online community.

Context	Participation Type	Method/Samples	Antecedents	Consequences	Source
Wikipedia: free online encyclopedia	Knowledge sharing	Survey/256 users	Trendsetting, opinion leadership, prosocial value orientation, intrinsic motivations	--	[19]
Douban: product review community	Level of participation	Secondary data/106 pages of book comment	Inclusiveness, reciprocity, centralization, centralization, core-periphery	Consumption intention	[37]
Seven automobile online communities	Product information sharing	Survey/283 users	--	Learning benefits, social benefits, self-esteem benefits, hedonic benefits	[20]
Mobile01: topic-free online community	Knowledge sharing	Survey/324 users	Reputation, reciprocity, enjoyment in helping others, knowledge self-efficacy, moderator’s enthusiasm, offline activities, enjoyability	--	[21]
--	Online participation	Literature review/--	Nature of the online community, individual characteristics, degree of commitment, quality requirement	--	[38]
470 online communities	On-topic discussion, off-topic discussion, generalized reciprocity, direct reciprocity,	Survey/1160 users	Common identity, common bond	--	[17]
Six online knowledge communities	Knowledge contribution	Survey/169 users	Perceived community support, perceived leader support	--	[12]
Online support communities focusing on pregnancy	Willingness to offer support	Survey/212 users	Action-facilitating support, nurturant support, self-efficacy, community identification	--	[39]
Online support communities for pregnant women	Online community citizenship behaviors	Survey/159 users	Subjective well-being, community identification	--	[16]
Facebook learning community	Knowledge-sharing behaviors	Survey/316 undergraduate students	Self-efficacy, sense of community	--	[26]
Bbs.feeyo.com: airline passenger online community	Knowledge sharing	Survey/364 users	Innovativeness, subjective knowledge, perceived ease of use, perceived usefulness	--	[22]
Smartphone’s online brand community	Product-information interaction, human-computer interaction, interpersonal interaction	Survey/665 users	--	Customer-brand relationship, customer-other customer relationship	[10]
Phoenix Health and Sweet Home: OHCs	General knowledge- sharing, specific knowledge-sharing	Survey/323 users	Sense of self-worth, face concern, reputation, social support, cognitive costs, execution costs	--	[7]
Good Doctor, Baby Tree, DXY: OHCs	Knowledge-sharing	Survey/443 users	Reputation, reciprocity, knowledge self-efficacy, altruism, empathy	--	[8]
Zhihu: social Q&A community	Knowledge contribution	Secondary data/3000 users	Identity-based trust, social feedback, identity communication, social exposure, norms of reciprocity	--	[18]
Zhihu: social Q&A community	Knowledge-sharing, knowledge integration	Survey/382 users	Knowledge self-efficacy, topic richness, Personalized recommendation, social interactivity	Community knowledge quality	[40]

**Table 2:** Research constructs and measurements.

Construct		Measurement Items	Sources
Perceived community support (PCS)		PCS1: The online health community provides various types of support for users to interact with each other, such as discussions, comments, reposting, likes, etc. PCS2: The online health community provides various modules to support user interactions on health and other topics PCS3: The online health community provides active users with proper rewards that represent their reputation or status	[7,11]
User interactions	Health topic interactions (HTI)	HTI1: When participating in the online health community, I usually actively share and discuss health information, treatment experience with others. HTI2: When discussing problems related to medical treatment and medical experience, I usually engage in subsequent interactions.	[7,17]
	General topic interactions (GTI)	GTI1: Members of the online health community interact actively with others on daily life and emotion-related topics. GTI2: Members in the online health community discuss actively public information, such as social, cultural, educational issues.	
Co-created value	Functional value (FV)	FV1: It allows me to increase my knowledge of the disease through members’ interactions FV2: It helps me solve problems associated with my disease through members’ interactions FV3: It helps increase my general knowledge besides health through members’ interactions	[7,20,39]
	Social value (SV)	SV1: I feel connected through members’ interactions in the online health community SV2: I can expand my social network through participation in community interactions. SV3: I can make friends in the online health community with whom I share common values or interests.	
	Affective value (AV)	AV1: I get comfort and care from other members through interactions AV2: I gain happiness through the interactions with other members AV3: I feel a sense of belonging through interactions with other members	
Continuous participation intention (CPI)		CPI1: I will continue participating in the community members’ interactions CPI2: I will take an active part in the discussions in this online health community CPI3: I will continue contributing my knowledge to other members and the community	[12,71]
Social exclusion (SE)		SE1: I feel uneasy when being with others because of my disease SE2: I feel lonely because I have no one to turn to SE3: People are around me but not with me	[40,44]

**Table 3:** Demographic characteristics of survey participants (*n* = 292).

Demographic Profile	Categories	Frequency	Percent (%)
Gender	Male	135	46.2
	Female	157	53.8
Age	Less than 25	21	7.2
	26–35	106	36.3
	36–45	117	40.1
	46 or above	48	16.4
Education	High school or below	99	33.9
	University college	169	57.9
	Graduate school	24	8.2
Monthly income (RMB)	Less than 3500	21	7.2
	3501–5000	77	26.4
	5001–8000	104	35.6
	8000 or above	90	30.8
Experience in this OHC	Less than 3 months	26	8.9
	3 months-1 year	138	47.3
	1 year or above	128	43.8

**Table 4:** Test results of internal reliability and convergent validity.

Construct	Items	Cronbach’s α	Convergent Validity		
			Factor Loading	Composite Reliability	Average Variance Extracted
PCS	PCS1	0.877	0.897	0.861	0.674
	PCS2		0.789		
	PCS3		0.772		
HTI	HTI1	0.872	0.887	0.872	0.773
	HTI2		0.871		
GTI	GTI1	0.773	0.697	0.782	0.646
	GTI2		0.898		
FV	FV1	0.928	0.914	0.933	0.823
	FV2		0.899		
	FV3		0.908		
SV	SV1	0.921	0.944	0.923	0.800
	SV2		0.879		
	SV3		0.858		
AV	AV1	0.913	0.915	0.913	0.778
	AV2		0.849		
	AV3		0.881		
CPI	CPI1	0.901	0.878	0.885	0.719
	CPI2		0.855		
	CPI3		0.810		
SE	SE1	0.901	0.917	0.904	0.759
	SE2		0.834		
	SE3		0.861		
χ^2^/df = 2.263, GFI = 0.905, RFI = 0.919, TLI = 0.953, CFI = 0.967, RMR = 0.036, RMSEA = 0.066					

**Table 5:** Mean, standard deviation, and correlation matrix.

Variables	Mean	SD	SCC	HTI	GTI	FV	SV	AV	CPI	SE
SCC	4.326	0.695	0.821							
HTI	3.938	0.941	0.436 **	0.879						
GTI	4.116	0.784	0.307 **	0.370 **	0.804					
FV	3.804	0.949	0.438 **	0.808 **	0.389 **	0.907				
SV	3.710	0.986	0.389 **	0.644 **	0.307 **	0.668 **	0.894			
AV	3.796	0.889	0.439 **	0.703 **	0.392 **	0.726 **	0.789 **	0.882		
CPI	3.693	0.868	0.538 **	0.767 **	0.406 **	0.811 **	0.688 **	0.754 **	0.848	
SE	4.457	0.707	0.736 **	0.191 **	0.325 **	0.192 **	0.273 **	0.376 **	0.324 **	0.871

Note. ** *p* < 0.01; the diagonal line of the correlation matrix represents the square root of AVE.

**Table 6:** Indirect effect of perceived social value on continuous participation intention.

Effect	Path	Standardized Estimate (β)	Unstandardized Estimate	Lower Bound	Upper Bound	*p*
Total effect	SV→CPI	0.226	0.174	0.118	0.231	0.001
Direct effect	SV→CPI	0.105	0.081	−0.027	0.187	0.122
Indirect effect	SV→AV→CPI	0.121	0.093	0.020	0.168	0.012
